# tDCS synchronous robotic training on lower-limb with post-stroke hemiplegia: a comparative study between synchronous and sequential group

**DOI:** 10.1080/07853890.2026.2722430

**Published:** 2026-08-25

**Authors:** Peishun Chen, Ju Sun, Yu Min, Wanrong Zhang, Taotao Li

**Affiliations:** Panyu Central Hospital, Guangzhou Medical University, Guangzhou, China

**Keywords:** Brain-limb coordinated regulation therapy, lower limb rehabilitation robot, tDCS, stroke, hemiplegia

## Abstract

**Background:**

This study was aimed to compare the effects of tDCS synchronized with robotic training(RT)and tDCS sequential followed by RT on lower-limb with post-stroke hemiplegia, thus to inform clinical decision-making between these delivery schedules.

**Methods:**

64 patients who met the inclusion and exclusion criteria were allocated into a synchronous group and a sequential group. The synchronous group underwent tDCS synchronous RT, while the sequential group received RT after tDCS ended. Both group received 40 minutes for treatment, five times a week, for a continuous two weeks. Gait Watch three-dimensional gait analysis system was used for both groups before and after treatment. Additionally, the Fugl-Meyer assessment for lower extremities (FMA-LE), functional ambulation category (FAC), and the modified Barthel index(MBI)were used to assess the motor function of lower extremities, walking function, and activities of daily living (ADL) in both groups.

**Results:**

Finally, 60 cases finished the study (30 for each group). Before treatment, there was no difference in baseline data between groups (*p* > 0.05). After treatment, there was no difference in FMA-LE between group (*F* = 1.751, *p* = 0.191). The incidence of 95% CI of the hip flexion, knee flexion, FAC, step difference and MBI in synchronous group were more superior than those in the sequential group (all *p* < 0.001).

**Conclusion:**

Synchronous tDCS-RT produced superior gait and functional benefits compared with sequential delivery, but did not differentially improve lower-limb motor impairment as measured by the FMA-LE on lower-limb with post-stroke hemiplegia.

## 1. Background and objectives

The incidence rate of stroke in China is high [1,2], most of which are accompanied by lower limb motor dysfunction [3]. Despite undergoing standardized rehabilitation therapy early on, numerous patients remain unable to regain walking ability or are left with severe gait abnormalities six months post-onset, profoundly affecting their daily activities and self-care abilities, and placing a considerable economic burden such as huge medical expenses and the substantial expenditure on medical insurance on both society and families [4].

For patients with post-stroke hemiplegia, regaining safe and functional walking is a central goal of rehabilitation because gait impairment directly affects mobility, independence, and quality of life.

Traditional rehabilitation methods most focus on peripheral stimulation [5], getting an uncertain efficacy. In recent years, brain regulation and stimulation technologies have witnessed rapid advancements [6–8]. Transcranial direct current stimulation (tDCS), as an emerging brain regulatory therapy technique, has been widely used in rehabilitation for stroke in recent years.

Robot training (RT) has been widely used in walking function rehabilitation in recent years. Although RT play a certain role on lower-limb motor function with post-stroke hemiplegia, they are mostly driven in a passive mode during the treatment process, with low active participation from the brain.

Combination therapy is a common method for disease rehabilitation. tDCS and RT are both effective methods for treating lower limb with stroke hemiplegia. However, there is still controversy on whether the two should be used synchronously or sequentially: (i)Select synchronization: Synchronous delivery may exploit use-dependent plasticity by pairing cortical priming with concurrent afferent input. tDCS-induced modulation of cortical excitability creates a transient plasticity window during which task-specific afferent input from RT may be more effectively consolidated. (ii) Select sequence: Sequential delivery may avoid potential interference or attentional division.

This study was aimed to compare the effects of tDCS synchronized with RT and tDCS sequential followed by RT on lower-limb with post-stroke hemiplegia, thus to inform clinical decision-making between these delivery schedules.

## 2. Methods

### 2.1. Inclusion and exclusion criteria

#### 2.1.1. Inclusion criteria

 

1. According to the diagnostic criteria for major cerebrovascular diseases in China, 2019 [9], patients were confirmed by cranial CT or MRI.
2. First-time onset, stable vital signs, disease duration of 2 weeks to 12 weeks, and age between 40 and 75 years.
3. The functional ambulation category (FAC) was Grade 1.
4. Minimum Mental State Examination (MMSE) ≥ 26 point.
5. Cerebral infarction or cerebral hemorrhage, with hemiplegia

#### 2.1.2. Exclusion criteria

 

1. Other neurological disorders.
2. Subarachnoid hemorrhage.
3. Bilateral stroke.
4. Bilateral limb dysfunction.
5. Other musculoskeletal or joint diseases (e.g. fractures, osteoarthritis) or neurological disorders (e.g. tremors, involuntary movements, Parkinson’s disease) affecting walking.
6. Patients who have undergone or are currently undergoing further brain stimulation therapies (e.g. transcutaneous magnetic stimulation) within the past 2 weeks.
7. Patients with contraindications for electrical stimulation therapy (e.g. pacemakers, local metal implants).
8. Severe concurrent diseases of the heart, liver, or kidneys.
9. History of cranial trauma, tumors, epilepsy, or a diagnosis of epilepsy.
10. Patients unwilling to enter into the informed consent form.

### 2.2. Sample size estimation

According to the formula for calculating the minimum sample size, N =(Z_α_+ Z_β_)^2^*2 S ^2^/𝛿 ^2^ [10], the sample size was estimated for the primary outcome (FMA-LE) using an expected between-group difference of 2.01 and standard deviation of 2.24 based on [11]. With alpha = 0.05, power = 0.9, and a 1:1 allocation ratio, the minimum required sample was 26 participants per group. After allowing for an anticipated dropout rate of 20%, the target enrollment was 32 participants. The final analysis included 64 participants.

### 2.3. Randomization and allocation

This study is a single-blind design and randomized controlled trial. The outcome assessors, data analysts were blinded after assignment to interventions. From February 1, 2024 to March 31, 2025, all 64 patients with stroke hemiplegia who met the acceptance criteria in the Affiliated Panyu Central Hospital of Guangzhou Medical University were selected as the research objects. The protocol was registered at the Chinese Clinical Trial Registry (ChiCTR2400080500), the registrational date was January 31, 2024, the date of initial patient enrollment was February 1, 2024.The dates of enrollment into the trial were February 1, 2024.

**Figure 1.**
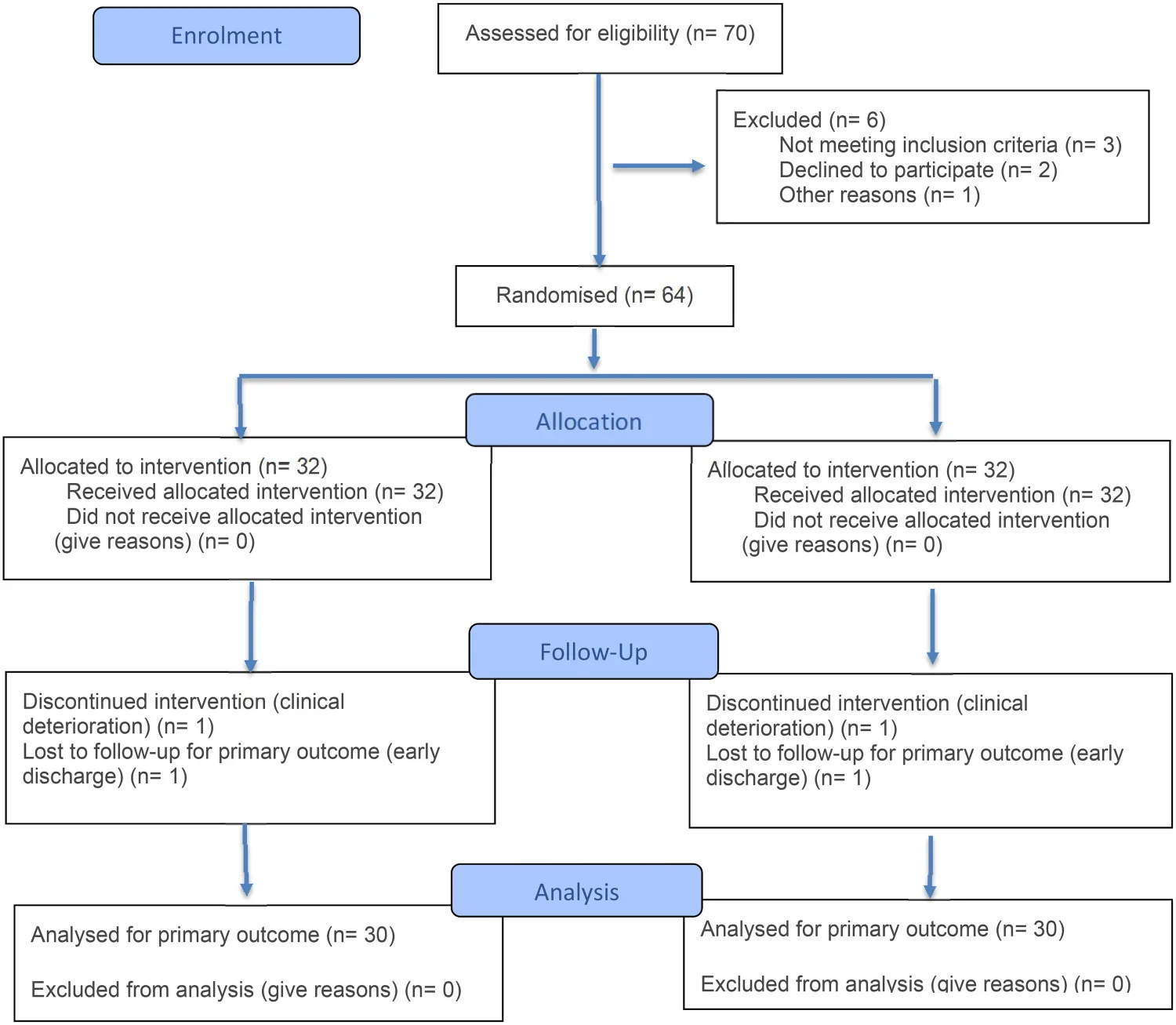
CONSORT 2025 flow diagram. Flow diagram of the progress through the phases of a randomised trial of two groups (that is, enrolment, intervention allocation, follow-up, and data analysis) [13].

The 64 enrolled patients were randomized into groups using the random envelope technique. The enrollment and assigned treatment allocation were handled by a clerk who was not involved in the evaluation and treatment. Specifically, 32 pieces of paper marked with ‘1” and another 32 marked with ‘2’ were placed into 64 identical opaque envelopes, which were then shuffled. Patients were allocated to either the synchronous group or the sequential group based on the number drawn from the envelope. All patients awared of the treatment plans for different groups and signed an attendance agreement to ensure their participation. All methods were performed in accordance with the applicable guidelines and regulations. The study had adhered to the principles stated in the ‘Declaration of Helsinki’ [12]. We have adhered to Consort guidelines [13] (Figure 1).

### 2.4. Intervention method

**Figure 2.**
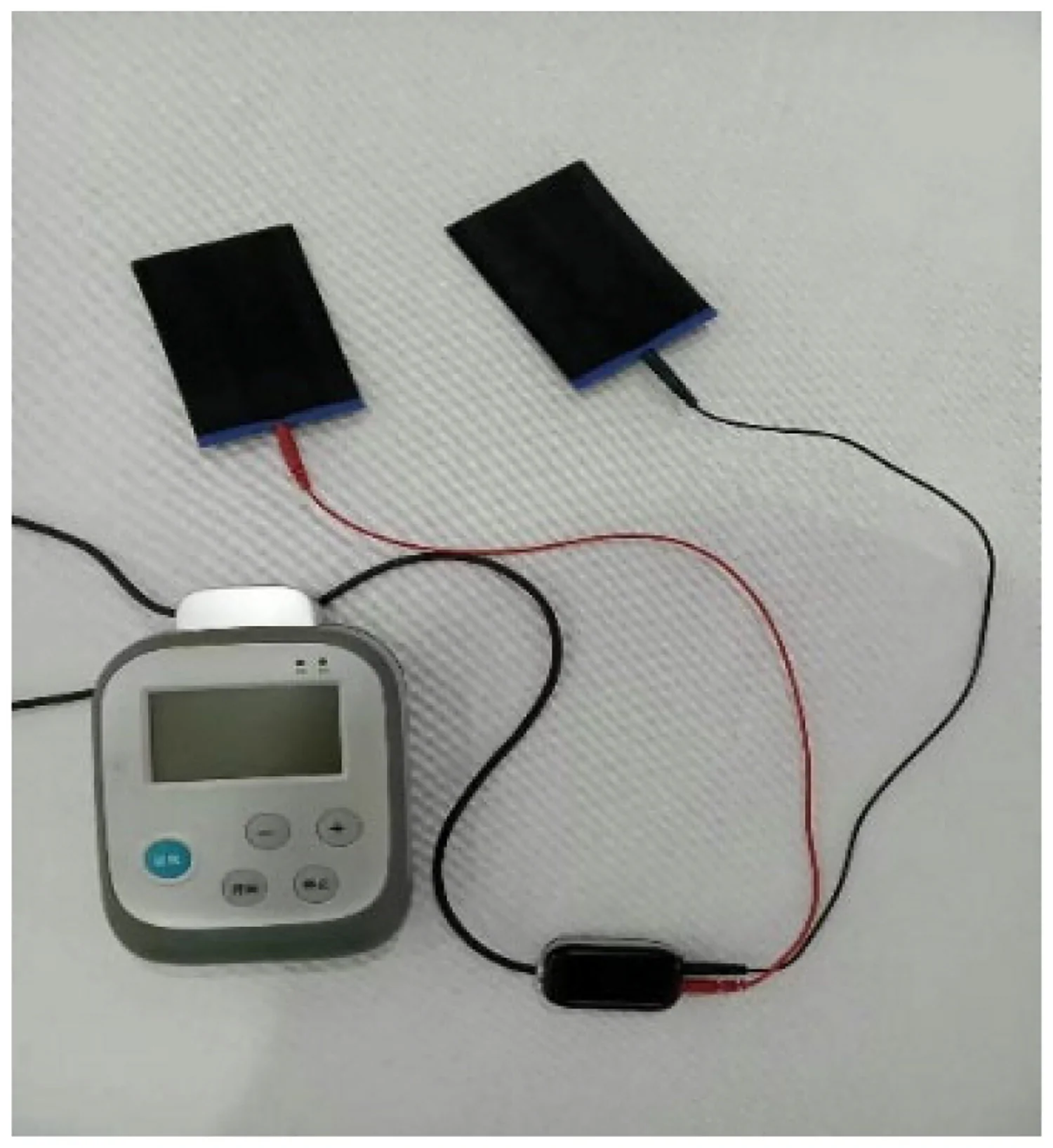
Transcranial direct current stimulation (tDCS).

**Figure 3.**
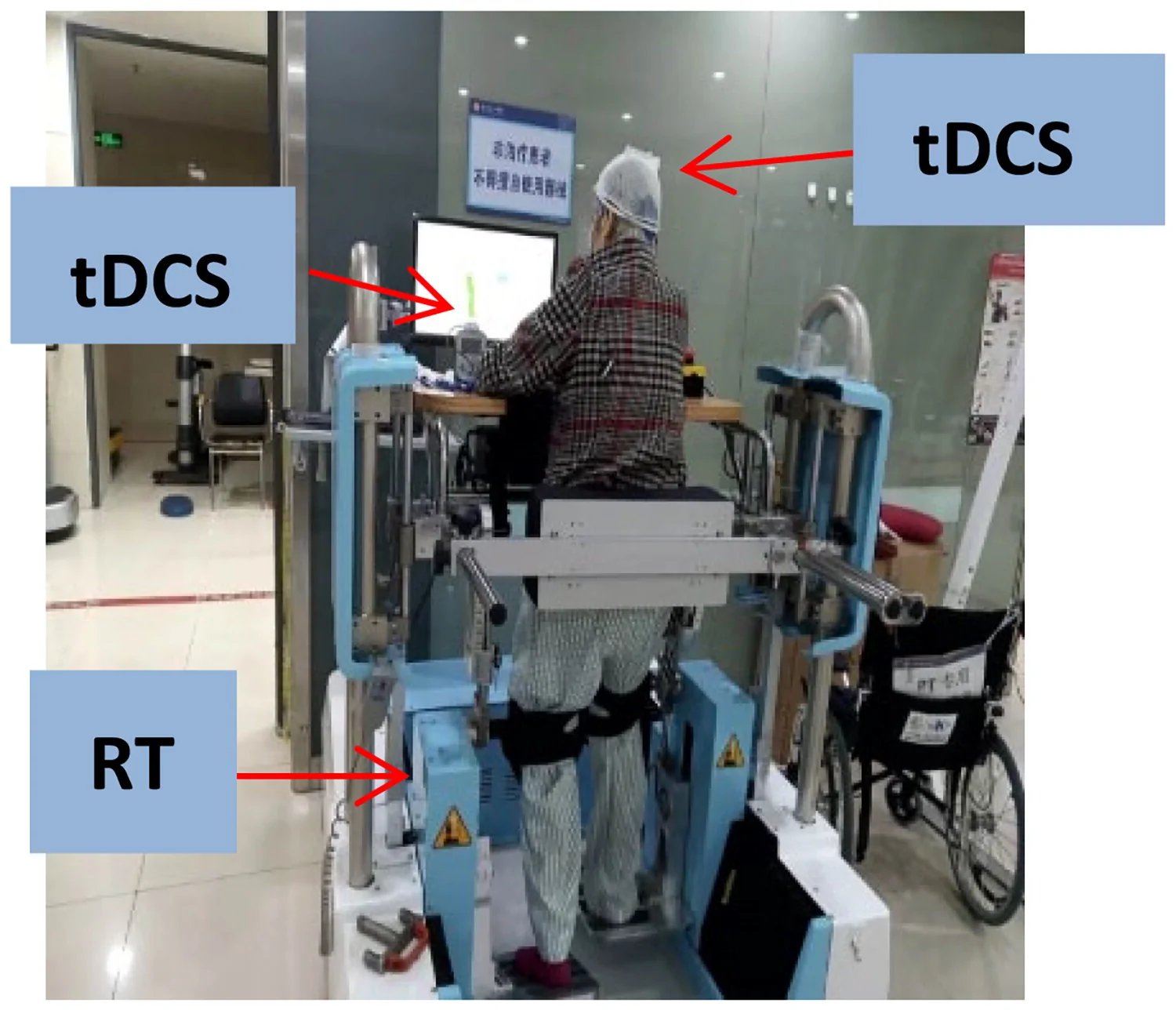
tDCS synchronous lower limb rehabilitation robot (RT).

tDCS or RT was operated by dedicated personnel respectively. The therapists were informed to perform tDCS (Figure 2) or RT (Figure 3) intervention. Both groups underwent conventional rehabilitation training, encompassing comprehensive training for hemiplegic limbs, motor control, balance function training, and walking exercises. Additionally, the synchronous group received synchronization therapy, using tDCS concurrently with RT (Figure 3), while the sequential group received sequential therapy, RT after tDCS ended. Both group received 40 min for treatment(The synchronous group received 20 min active tDCS concurrent with 20 min of RT, then 20 min of sham tDCS which used the standard 30-second ramp-up/ramp-down to mimic the initial tingling sensation. The sequential group received 20 min active tDCS followed by 20 min of RT). The total active tDCS dose is therefore matched, but the total session duration differs (40 min in both), five times a week, for a total of two weeks.

**Figure 4.**
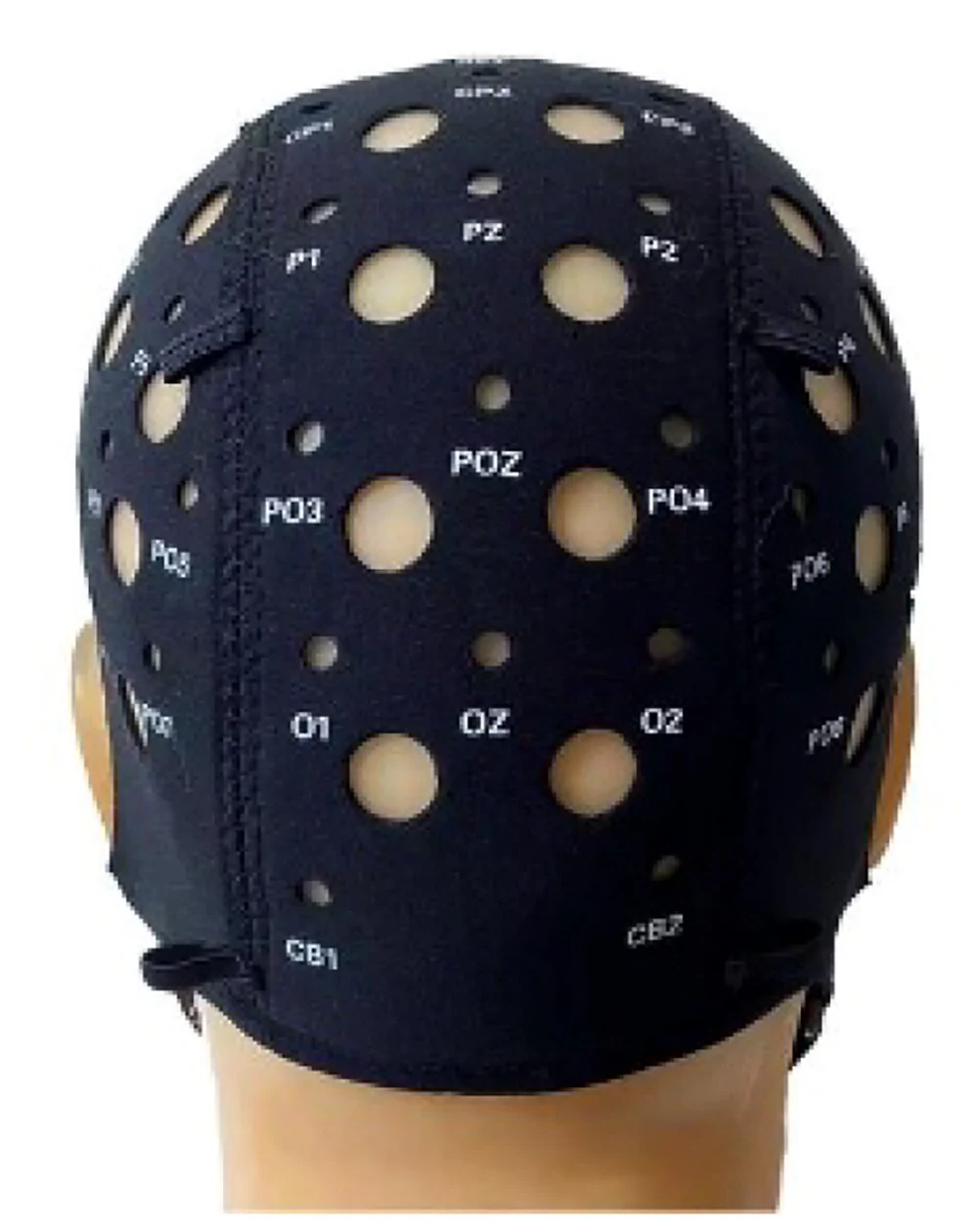
Positioning cap.

**Figure 5.**
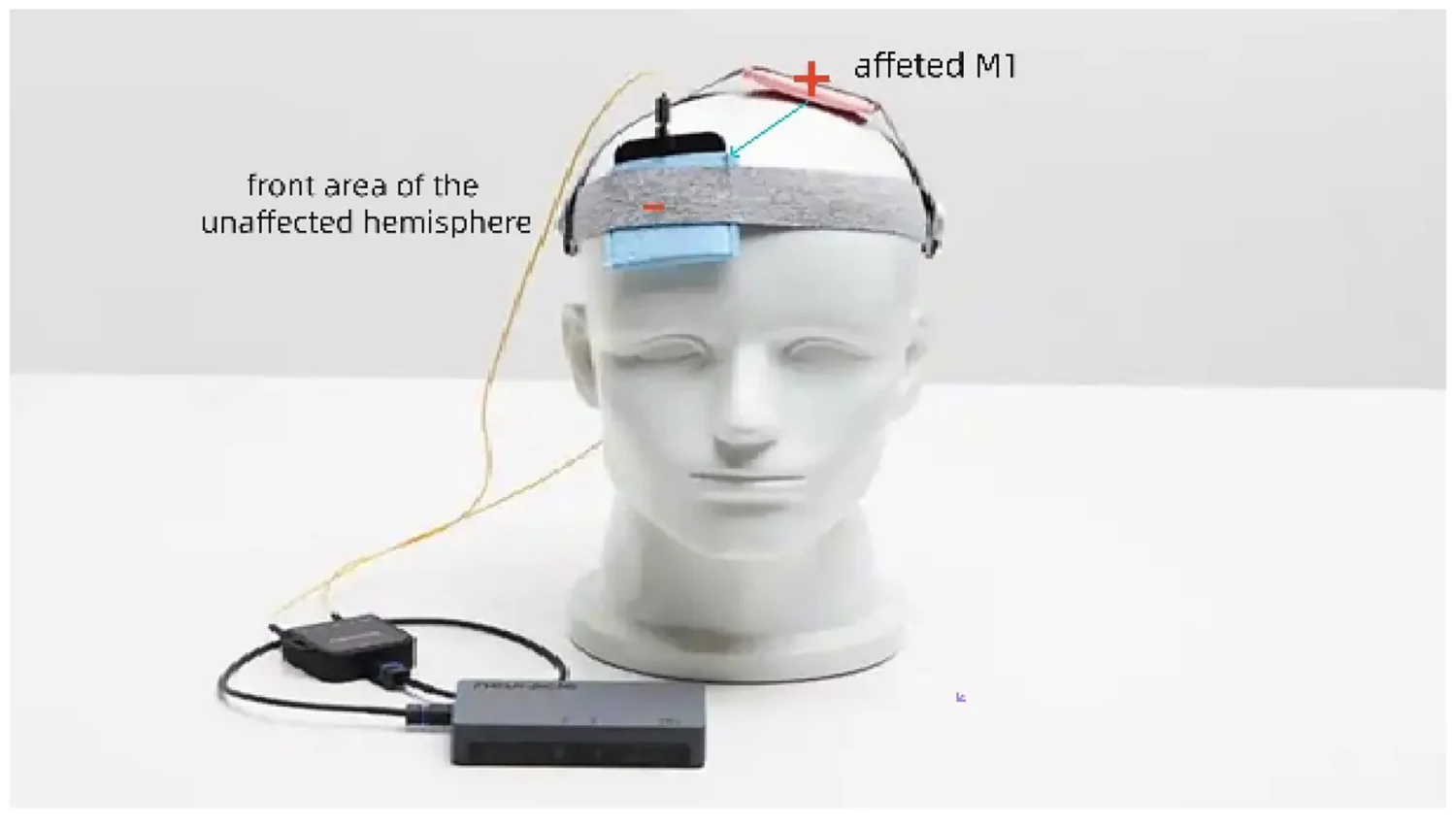
Direction of electric current.

The tDCS (IS300 intelligent electrical stimulator, made in Sichuan, China) has both anode and cathode electrodes measuring 5 cm × 7cm. The anode was positioned on the affected M1 area (Positioning method: Wearing a positioning cap (Figure 4) designed according to the EEG 10–20 system, with the left M1 area corresponding to the C3 electrode position and the right side corresponding to the C4 electrode position) of the affected hemisphere, and the cathode was placed on the frontal area of the unaffected hemisphere. The current flows from the positive pole to the negative pole (Figure 5). The current intensity was set at 2 mA [14], the current density was 0.057 mA/cm^2^ and total delivered charges were 2 mA, current ramp-up/ramp-down (5–10 s), which with a slight numbness in the stimulated area.

The RT (Model MRG-P100 rehabilitation robot, made in Taiwan), selecting the ‘gait sensation input’ which used an active-assisted mode with a step distance of 35 cm, a step frequency of 25 steps per minute (the device specification, one mode corresponds to one parameter), and a training time of 20 min. During the treatment, the patients were walking with body-weight supported by the exoskeletons of RT.

Ultimately, ten adverse events occurred which were mild during the therapy. The synchronous group got 2 skin irritation, 1 headache and 2 tingling. The sequential group got 2 skin irritation, 2 headaches and 1 tingling. The symptomatic was mild and does not affect subsequent treatment. There were no serious adverse events in either group. There was no significant difference in the incidence of adverse events between the two groups.

### 2.5. Data collection

Before treatment (When preparing to receive the first treatment protocol) and after treatment (After completing implementation of implemented protocol), both groups underwent analysis using the ZhangHe Gait Watch 3D gait analysis system to assess parameters such as hip and knee flexion angles of the affected lower limb [15], and step length differences between the left and right sides. Furthermore, the Fugl-Meyer Assessment for lower extremities (FMA-LE, 0–34 points, the higher the score, the better the motor function), Functional Ambulation Category (FAC, level 0, level 1, level 2, level 3, level 4 and level 5 were, respectively, recorded as 0, 1, 2, 3, 4 and 5 grades. The higher the grade, the greater the walking ability), and Modified Barthel Index (MBI, 0–100 points, the higher the score, the higher the daily living ability) were employed to evaluate motor function, walking capacity, and daily living activities of the affected lower limb in both groups.

**Figure 6.**
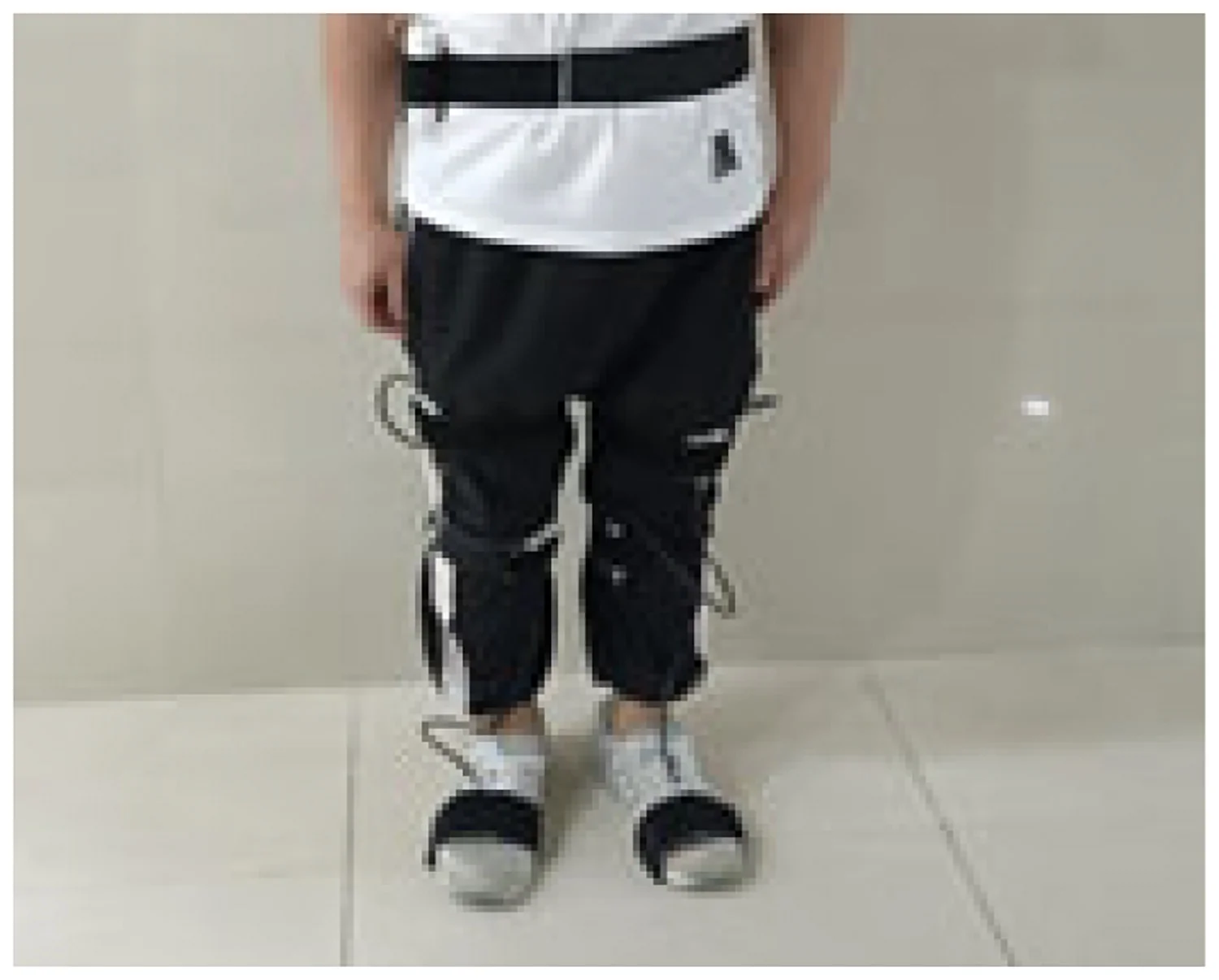
Gait watch.

Gait Watch (Guangzhou ZhangHe, made in China, Figure 6) system is a 3D motion capture system used for gait analysis, mainly applied in clinical research and rehabilitation training evaluation. When measuring, the bluetooth sensor is worn near the hip, knee, ankle and other joints, and the patient is allowed to walk in a straight line for a distance of 12 meters. Gait Watch software can be used to measure gait parameters (step length, stride length, step speed, joint flexion angle, support time, etc.).

### 2.6. Statistical methods

Statistical analysis was performed using SPSS 29.0. Perform normality test was done on the obtained data. For within-group comparisons, normally distributed measurement data was presented as mean ± standard deviation with independent sample T-test used, non-normally distributed measurement data was presented as median (Q1, Q3) with rank-sum tests used, count data was presented as number of cases (%) with Chi-square used. For between -group comparisons, ANCOVA test was used, the ANCOVA model was baseline score. The assumption of homogeneity of regression slopes was tested. Rank ANCOVA test was use for non-normally distributed data. The inspection level is set to α = 0.05, *p* < 0.05 indicates a statistically significant difference. We used complete-case analysis to handle the lost excluding dropouts and a sensitivity analysis under intention-to-treat with multiple imputation.

## 3. Results

### 3.1. Comparison of baseline characteristics between the synchronous group and sequential group

Before treatment, there were no statistically significant differences between the two groups in terms of gender, age, disease course, disease type, hip flexion angle, knee flexion angle, step difference, FAC, FMA-LE and MBI (all *p* > 0.05) (Table 1).

**Table 1.** Baseline data between synchronous group and sequential group.

Baseline data	Synchronous group (*n* = 30)	Sequential group (*n* = 30)	*X^2^*/*t*	*p*
**Sex** [man/feman, n(%)]	18(60.00)/12(40.00)	19(63.33)/11(36.67)	0.071	0.791
**Age**(Years, $\bar{x}\pm s$ )	62.67 ± 14.10	63.03 ± 12.48	−0.107	0.915
**Course**(day, $\bar{x}\pm s$ )	58.47 ± 18.79	57.80 ± 19.05	0.136	0.892
**Type** [hemorrhage / infarction, n(%)]	11(36.67)/19(63.33)	11(36.67)/19(63.33)	0.000	1.000
**Hip flexion**(°)	10.87 ± 4.04	10.37 ± 4.12	0.474	0.637
**Knee flexion**(°)	31.30 ± 10.51	31.03 ± 10.32	0.099	0.921
**Step difference**(cm)	11.97 ± 2.89	11.03 ± 3.11	1.203	0.234
**FAC** (Grade)	1.10 ± 0.31	1.07 ± 0.25	0.460	0.647
**FMA-LE** (Score)	13.57 ± 2.45	12.43 ± 2.13	1.915	0.061
**MBI**(Score)	69.07 ± 4.61	67.27 ± 4.60	1.514	0.135

Above all, *p* > 0.05, FAC: Functional Ambulation Category; FMA-LE: Fugl-Meyer Assessment for lower extremities; MBI: Modified Barthel Index.

### 3.2. Primary/secondary outcomes

Primary = FMA-LE; Secondary = FAC, MBI, gait

#### 3.2.1. Primary outcomes

**Figure 7.**
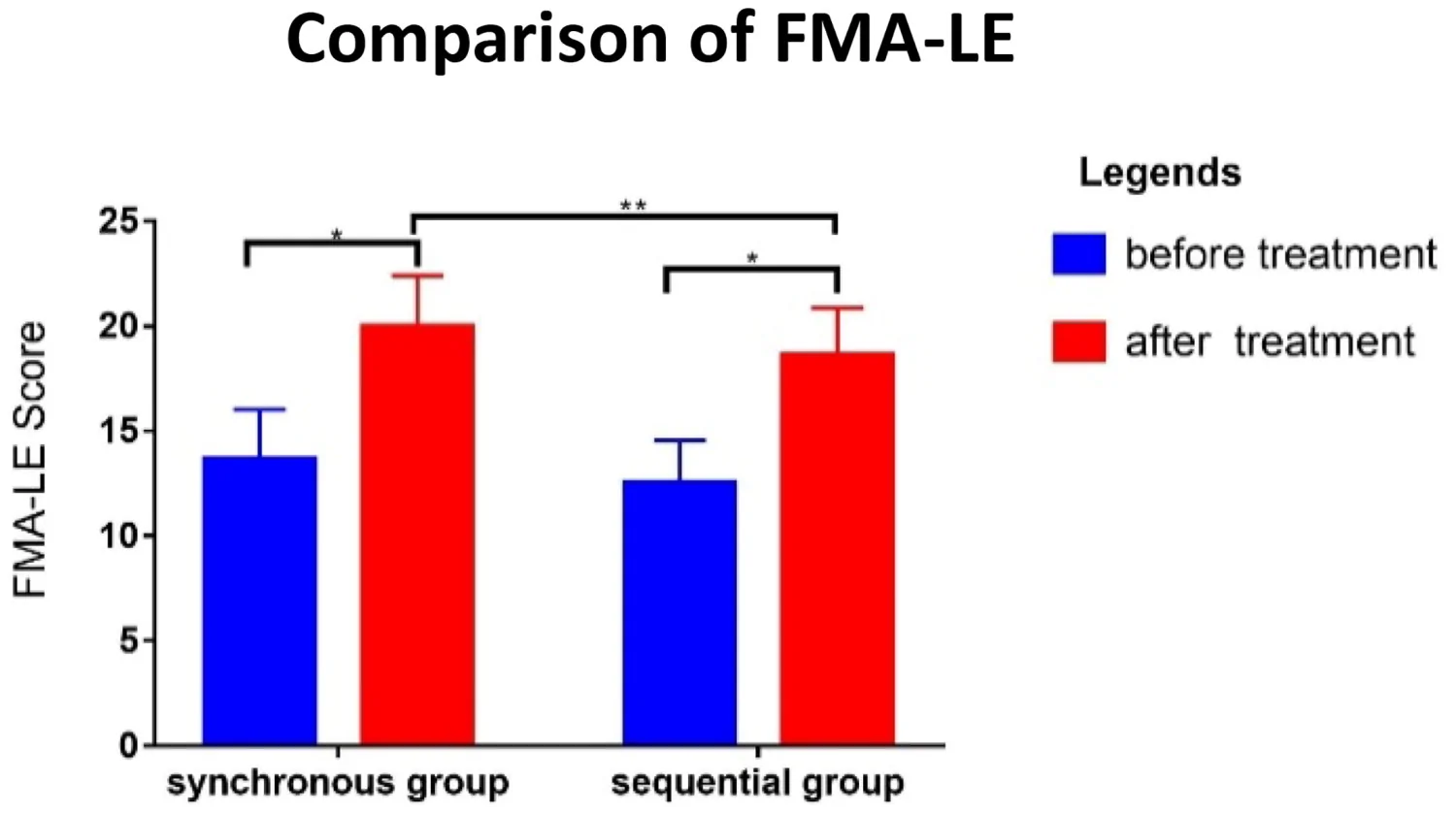
Mean FMA-LE Score (±1 standard deviation) of two group before and after treatment, with the horizontal axis representing grouping and the vertical axis representing FMA-LE Score, within-group, *p* < 0.001, between-group, *p* > 0.05, FMA-LE: Fugl-Meyer Assessment for lower extremities.

After treatment, the synchronous group 95% CI (18.96, 20.84), sequential group 95% CI (17.66, 19.40), the MD(95% CI)was 1.367 (0.115, 2.618), there was no difference between group (*F* = 1.751, *p* = 0.191). This may be related to the short observation time or the same stimulation done on lower limb in two group. There was significant difference within group in FMA-LL, synchronous group (t = −15.321, *p* < 0.001), sequential group (t = −14.560, *p* < 0.001) (Figure 7).

#### 3.2.2. secondary outcomes

**Figure 8.**
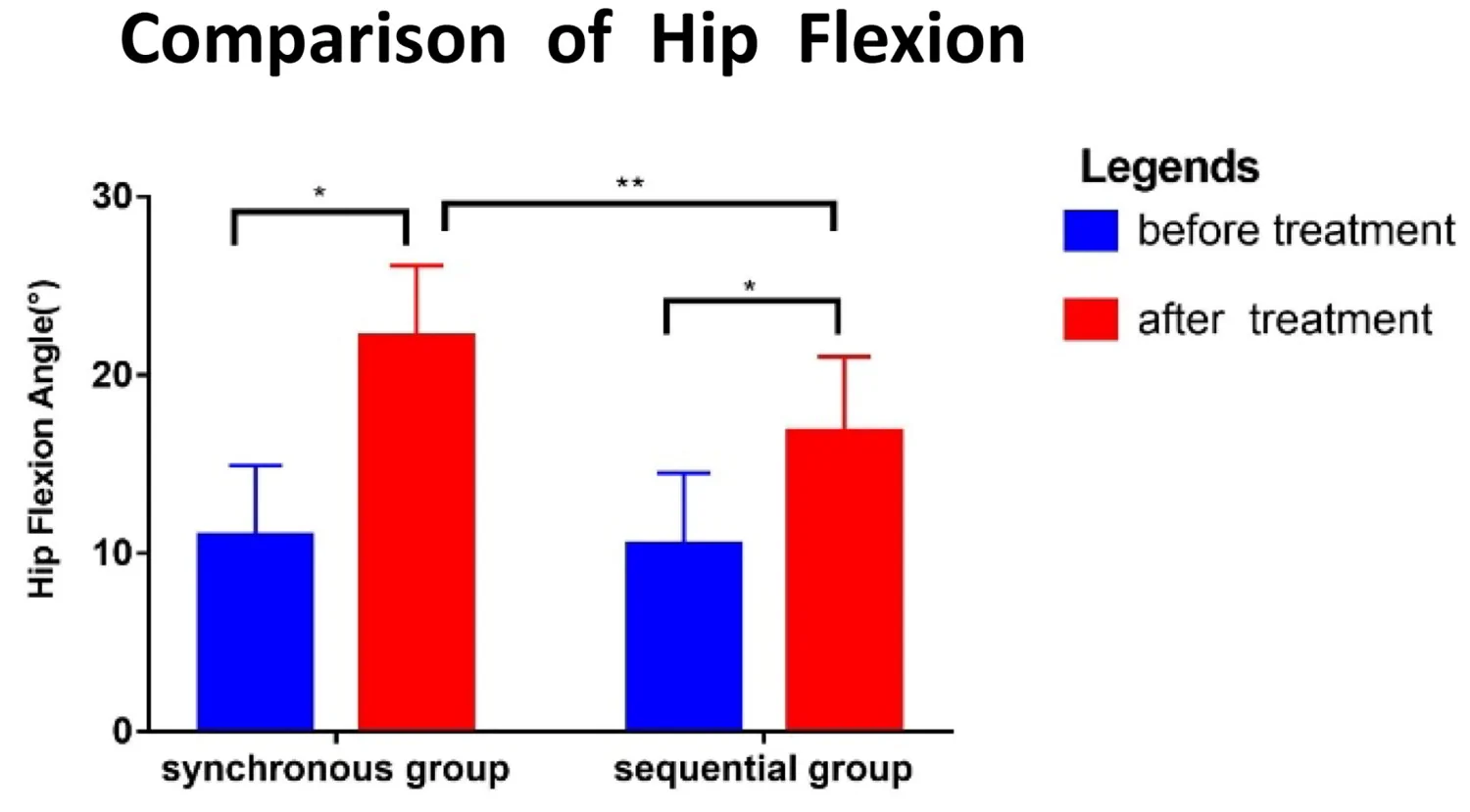
Mean hip flexion angle (±1 standard deviation) of two group before and after treatment, with the horizontal axis representing grouping and the vertical axis representing Hip Flexion Angle, within-group, *p* < 0.001, Between-group, *p* < 0.001.

**Figure 9.**
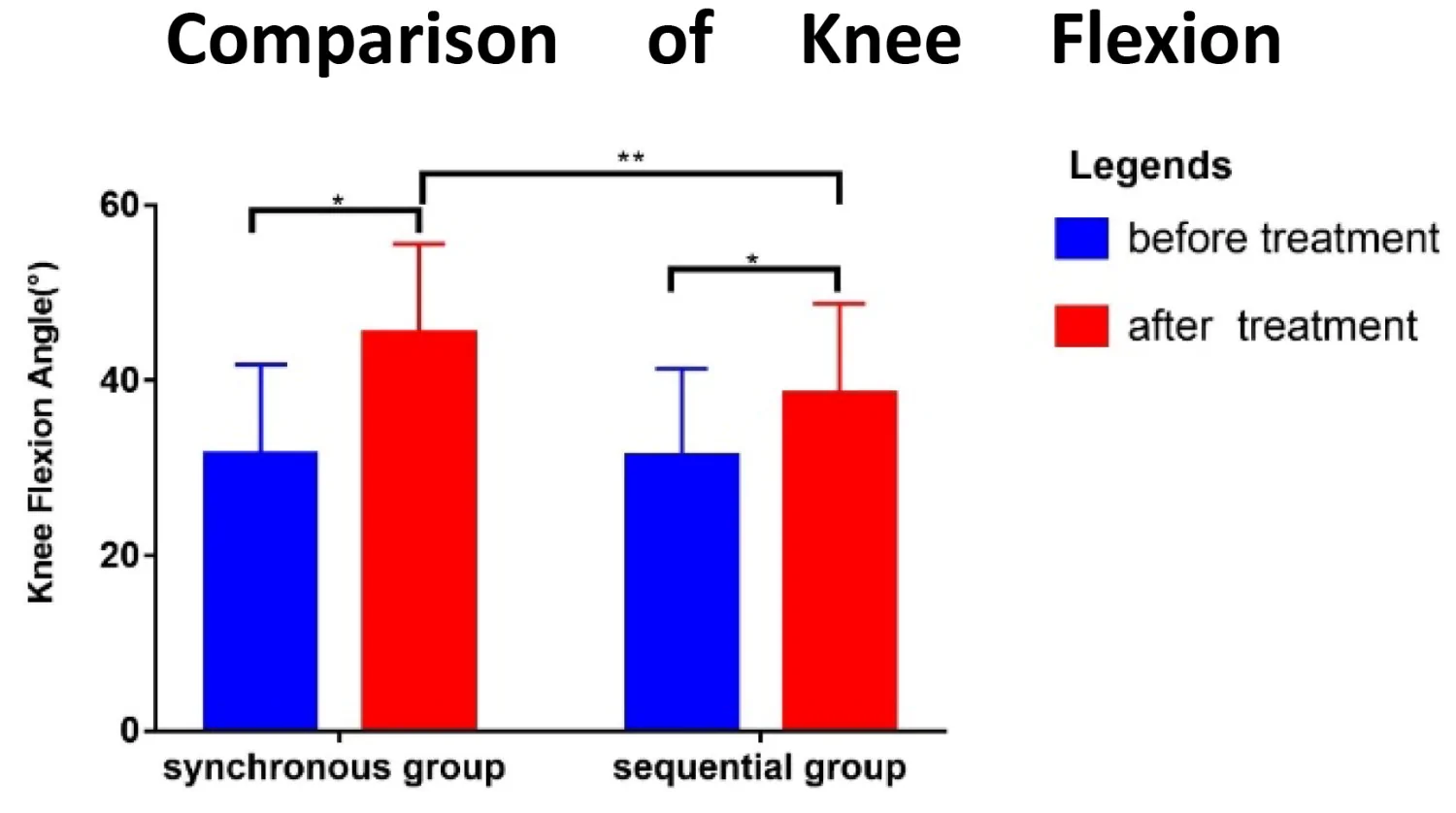
Mean knee flexion angle (±1 standard deviation) of two group before and after treatment, with the horizontal axis representing grouping and the vertical axis representing Knee Flexion Angle, Within-group, within-group, *p* < 0.001, between-group, *p* < 0.001.

**Figure 10.**
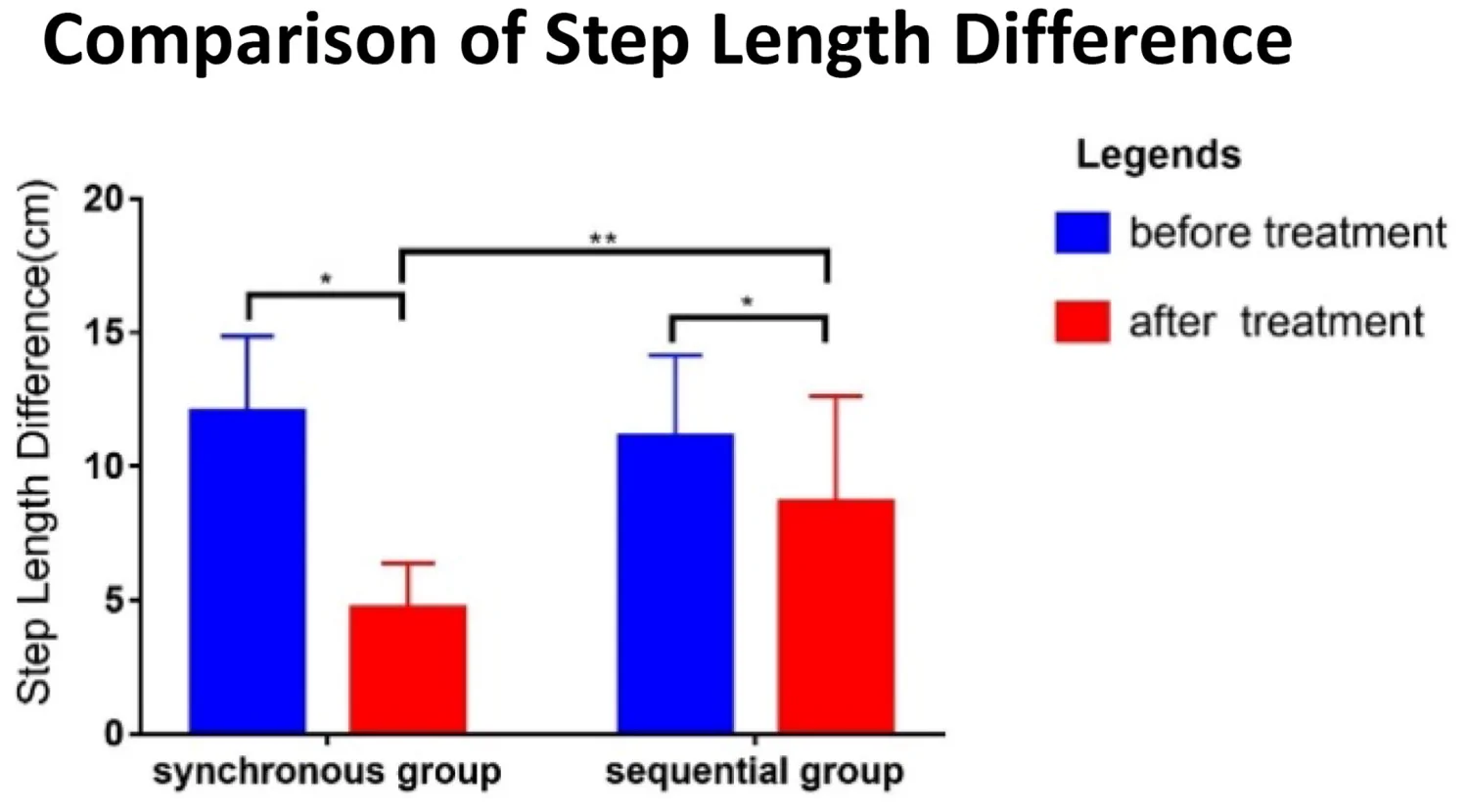
Mean step length difference (±1 standard deviation) of two group before and after treatment, with the horizontal axis representing grouping and the vertical axis representing Step Length Difference, within-group, *p* < 0.001, between-group, *p* < 0.001.

**Figure 11.**
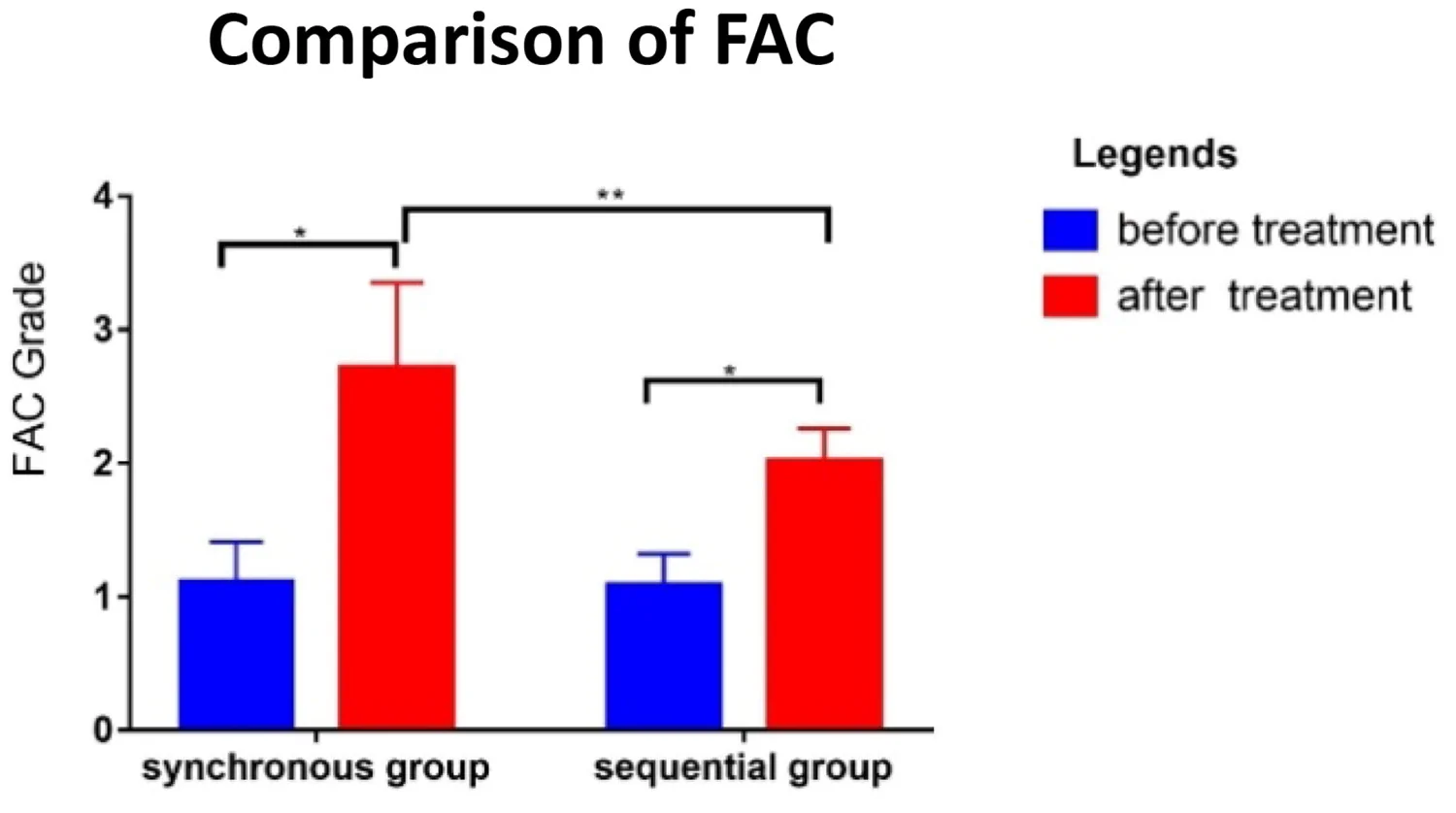
Mean FAC grade (±1 standard deviation) of two group before and after treatment, with the horizontal axis representing grouping and the vertical axis representing FAC grade, within-group, *p* < 0.001, between-group, *p* < 0.001. FAC: Functional Ambulation Category.

**Figure 12.**
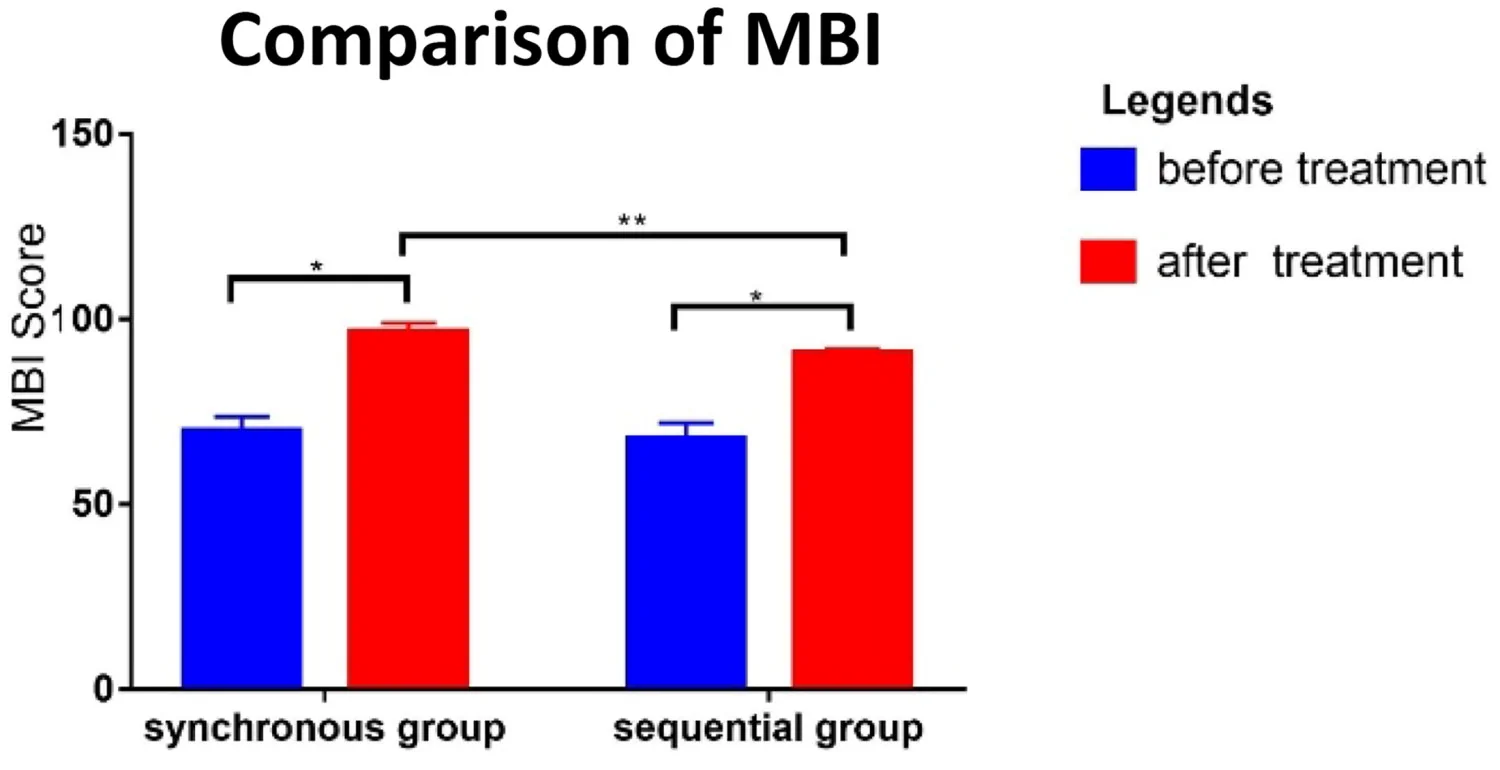
Mean MBI score (±1 standard deviation) of two group before and after treatment, with the horizontal axis representing grouping and the vertical axis representing MBI Score, within-group, *p* < 0.001, between-group, *p* < 0.001. MBI: Modified Barthel Index.

After treatment, there were significant differences within group in hip flexion, knee flexion, step length difference, the functional ambulation category, and modified Barthel index (all *p* < 0.001). The synchronous groups demonstrated superior to the sequential groups in all aforementioned indicators (all *p* < 0.001) (Table 2 and Figures 8–12).

**Table 2.** Comparison of secondary outcomes.

Team	Hip flexion (95%CI, °)	Knee flexion (95%CI, °)	Step difference (95%CI, cm)	FAC (95%CI, Grade)	MBI (95%CI, Score)
**Synchronous group (*n* = 30)**					
Before treatment	(9.36, 12.38)	(27.38, 35.22)	(10.89, 13.05)	(0.99, 1.21)	(67.35, 70.79)
After treatment	(20.50, 23.57)	(41.23, 49.04)	(3.98, 5.29)	(2.46, 2.94)	(95.03, 97.17)
*t*	−16.303	−31.845	19.530	−4.920	−55.969
*p*	<0.001	<0.001	<0.001	<0.001	<0.001
**Sequential group (*n* = 30)**					
Before treatment	(8.83, 11.91)	(27.18, 34.89)	(9.87, 12.20)	(0.97, 1.16)	(65.55, 68.98)
After treatment	(15.09, 18.31)	(34.31, 42.16)	(7.39, 9.74)	(1.90, 2.10)	(89.56, 90.84)
*t*	−16.342	−27.236	12.577	−4.596	−5.245
*p*	<0.001	<0.001	<0.001	<0.001	<0.001
MD (95% CI)	5.333 (3.156, 7.511)	6.900 (1.482, 12.318)	−3.933 (−5.250, −2.617)	0.700 (0.443, 0.957)	5.900 (4.683, 7.117)
*F*	57.527	167.926	26.223	35.022	142.444
*p*	<0.001	<0.001	<0.001	<0.001	<0.001

Comparison within groups before and after treatment, *p* < 0.001; Comparison between groups after treatment, *p* < 0.001, FAC: Functional Ambulation Category; MBI: Modified Barthel Index.

## 4. Discussion

tDCS is a non-invasive brain stimulation technique characterized by its non-invasiveness, convenience, cost-efficiency, and minimal side effects. There is study showed that it can effectively modulate cortical excitability [16]. By administering low-intensity, unvarying direct current stimulation to the brain, it addresses interhemispheric inhibitory imbalance by either enhancing excitability in the affected hemisphere or diminishing it in the healthy hemisphere [17]. Specifically, the anode elicits an excitatory effect, whereas the cathode exerts an inhibitory influence [18]. Research conducted by Miranda de Aquino Miranda J and Xu Q [19,20] has demonstrated that tDCS combined with task-oriented training can augment lower-limb motor function and balance in patients with hemiplegic recovering from stroke or traumatic brain injury, notably enhancing their walking capabilities. tDCS boasts advantages such as high safety, portable equipment, and prolonged aftereffects, rendering it widely integrated with limb rehabilitation techniques. In summary, tDCS has a positive effect on the recovery of lower limb with post-stroke hemiplegia.

Robots training (RT) represent automated rehabilitation training devices that harness the principle of motor relearning to engage patients in repetitive, intensive, and task-oriented movements. There is study showed that it can faster gait functional recovery [21]. Some study showed that it can exhibit beneficial effects on enhancing balance control, walking ability (encompassing gait speed, walking distance, symmetry, and coordination), and rectifying abnormal muscle tone in patients with acute and subacute strokes [22]. Studies by Chen Fang and Chang [23,24], among others, have revealed that training with ground-walking lower-limb exoskeleton robots can improve walking ability and lower limb motor function in stroke patients. Wu Yuefeng et al. conducted a study to observe the clinical efficacy of combining scalp acupuncture therapy with lower-robot-assisted gait training in improving abnormal gait among stroke patients through three-dimensional gait analysis. They discovered that after treatment, stride length, walking speed, step width, and percentage of double-limb support were significantly improved compared to pre-treatment levels [25]. Similarly, Kim Y et al. [26] discovered that lower-limb rehabilitation robot training can enhance lower-limb muscle strength in stroke patients during the recovery phase, ameliorate abnormal gait parameters, elevate motor and balance functions, and augment walking ability, advocating for its extensive application in rehabilitation therapy. In summary, robot training has a positive effect on the recovery of lower limb with post-stroke hemiplegia.

The results of this study suggest that after treatment, synchronous group and sequential group showed improvements in FAC, MBI, and gait parameters compared to before treatment. This is related to the fact that both tDCS and RT can promote the rehabilitation of lower limb with stroke hemiplegia. With the improvement of walking function, gait parameters, and ADL will also improve.

After the treatment, the synchronous group demonstrated superior to the sequential group in FAC, MBI, and gait parameters. It shows that the synchronous treatment of tDCS and RT is more effective than sequential treatment. So, the possible mechanism will be follows: (i) Anodic excitation: The excitation of the motor cortex by tDCS anodes is consistent with task related input of RT, and synaptic connections are strengthened through spike time dependent plasticity (STDP) [27]. (ii) Neural mechanisms: By inhibiting the healthy side motor cortex through tDCS cathodes, it promotes balance between the bilateral cerebral hemispheres [28]. Synchronized RT promotes neural plasticity in the affected side. (iii) Neurovascular coupling [29]: The neurovascular coupling increased by tDCS and RT. (iv) Enhanced brain functional connectivity: Studied by Li Ce et al. [30] showed that tDCS can regulate brain functional connectivity. Because tDCS can strengthen brain functional connectivity, positive effect on the recovery of lower limb with post-stroke hemiplegia, tDCS synchronous RT can promote brain limb synergy and better facilitate the recovery of lower limb with stroke hemiplegia. However, these are hypotheses and no neurophysiological or imaging data were collected to test them directly. The relevant mechanisms need further research.

Combination therapy is a common method for disease rehabilitation. ‘Brain-limb coordinated regulation therapy’ [31] means activating brain regions combine with limb task training. It integrates central nervous and peripheral intervention techniques, which was being investigated and implemented in clinical practice. In this study, lower-limb rehabilitation robots were used to train the limbs, while tDCS was concurrently applied to the affected side of the brain in stroke patients. The findings revealed that the hip flexion angle and knee flexion angle of the affected lower-limb in the synchronous group were greater than those in the sequential group. Additionally, the difference in step length between the left and right limbs was smaller in the synchronous group compared to the sequential group. Furthermore, the lower limb walking function classification, and modified Barthel index were all higher in the synchronous group than in the sequential group (*p* < 0.05). However, there was no difference between the two groups (*p* > 0.05). This may be related to the short observation time or the same stimulation done on lower limb in two group. This indicates that tDCS synchronous RT can more effectively improve the gait and functional benefits. With the augmentation of hip and knee flexion angles, walking performance improved, walking ability was strengthened, and gait became more symmetrical. Therefore, after treatment, the step length difference between the left and right limbs decreased, and the walking function classification was improved. With the enhancement of walking ability, patients find it more convenient to engage in daily living activities, subsequently resulting in an increase in the modified Barthel index scores. However, the sequential group of tDCS and RT were separated, and the regulation of the brain by tDCS could not form a closed loop stimulation with the treatment of the lower limbs by RT, so the effect was not as good as that of the synchronous group. Bernal-Jiménez JJ et al. [32] discovered that combining bilateral transcranial direct current stimulation with robot-assisted treatment did not yield additional improvements in FMA-LE score, functional independence measure, or action research arm Test scores among stroke patients. It is similar to the results of this study. Both domestic and international studies have corroborated that ‘brain-limb synergy’ therapy outperforms single rehabilitation techniques in improving walking functions, such as gait speed and gait parameters, for stroke patients with walking impairments [33]. With the regulation of tDCS, RT can better promote the rehabilitation of lower-limb movement in stroke hemiplegia, thereby improving different outcomes. This may be related to the synergistic effect of tDCS and RT in treatment. There are researches which demonstrated that tDCS can regulate the dynamic brain network connections in stroke patients with motor dysfunction, and facilitate brain functional reorganization [34–36].

In summary, synchronous tDCS-RT produced superior gait and functional benefits compared with sequential delivery, but did not differentially improve lower-limb motor impairment as measured by the FMA-LE on lower-limb with post-stroke hemiplegia, and that these preliminary findings warrant confirmation in larger, sham-controlled trials with neurophysiological and imaging correlates.

This study has its limitations, (i) the single-blind design and lack of sham tDCS in the sequential group; (ii) the absence of neuroimaging or neurophysiological correlates; (iii) the short 2-week intervention and lack of follow-up beyond the immediate post-treatment assessment; (iv) the restriction to FAC Grade 1 patients, which limits generalisability; and (v) the absence of a no-tDCS or sham-tDCS control arm, which precludes disentangling the specific contribution of synchronous timing from the general benefit of combined therapy.

Therefore, synchronous delivery may be preferred for gait and functional outcomes, but that this requires confirmation in adequately powered sham-controlled trials with longer follow-up.

## Data Availability

All data are included in this manuscript. The data that support the findings of this study are available from the corresponding author upon request. The corresponding author is Wanrong Zhang (E-mail: 28267864@qq.com).
